# The role of sexual compulsivity in unprotected intercourse among STI patients in Shanghai, China

**DOI:** 10.1186/s12889-021-10186-0

**Published:** 2021-01-15

**Authors:** Yang Ni, Hengan Liu, Ruijie Gong, Mei Shi, Shuxian Zhang, Suping Wang, Yong Cai

**Affiliations:** 1 Shanghai Skin Disease Hospital, Shanghai, 200443 China; 2 School of Public Health, Shanghai Jiao Tong University School of Medicine, Shanghai, 200025 China; 3 Shanghai Xuhui Center for Disease Control and Prevention, Shanghai, 200237 China; 4 Shanghai Jiao Tong University, School of Medicine, Shanghai, 200025 China

**Keywords:** Sexually active, Unprotected intercourse, Sexually compulsivity, Sexually transmitted infections, Patients

## Abstract

**Background:**

Sexual compulsivity (SC) and its relationship with unprotected intercourse (UI) have long been an intriguing topic, but its existential meaning in the management of public health or, more precisely, sexually transmitted infections (STIs) has rarely been studied to date. This study examines whether SC plays a role in UI among sexually active STI patients.

**Method:**

A cross-sectional study was conducted in two sexual transmitted disease (STD) clinicals of Shanghai Skin Diseases Hospital in Shanghai. Totally 664 sexually active STI patients were included.

**Results:**

The ages of the 664 participants ranged from 18 to 76 years, with 58.73% between 26 and 40 years old. 449 (191 male and 258 female) reported had UI during the past 6 months. Although the only statistically significant difference (*p* < 0.01) was in relation to UI with a casual sexual partner, the difference between male/female and regular/casual sexual partners remained evident.

**Conclusions:**

SC is evidently a potential predictor of UI with a casual sexual partner in male STI patients, while the use of condoms is more likely to be affected by other factors. In addition to general sexual education, counseling interventions should be provided by health institutions, and specific intervention methods targeting gender and sexual partners should be considered.

## Background

Unprotected intercourse (UI) has long been proved to be a risk factor for the spread of sexually transmitted infections (STIs) including human immunodeficiency virus (HIV) infection [1, 2], unintended pregnancy, and abortion [3, 4], especially in sexually active populations. Approaches such as small-group enhanced counseling [5], condom promotion interventions [4, 6], and cognitive behavioral interventions [7] have been taken to prevent UI and yield diverging results around the world. A correlation between sexual compulsivity (SC) and UI has been found in several studies [8–10] but has lacked attention from researchers and is worthy of further study. SC is defined as an insistent, repetitive, intrusive, and unwanted urge to perform specific acts often in ritualized or routinized fashions, which involves a focused and intense necessity to meet sexual needs [11]. The extent to which said compulsivity can be treated as a behavioral pattern at the extreme of the normal range, or should be considered a behavioral pattern deviating from the norm, is not fully understood [12].

Over the past decades, several studies have focused on the role of SC in UI among specific groups [8–10, 13–18]. Studies concerning the HIV-positive population dating back to the 1990s found that high SC, measured with the Sexual Compulsivity Scale (SCS) created by Kalichman in 1994 [11], can be associated with UI. Men who have sex with men (MSM) was also a popular focus among researchers, with most studies producing similar results suggesting that sexually compulsive participants are more likely to take part in risky sexual behavior, especially UI [14, 16]. Research targeting other populations such as male and female college students [17], female migrant workers [18], heterosexual males [10], and other specific groups has been published. Most studies found a clear correlation between high SC and UI. Currently, although UI presents the greatest sexual risk for STIs, to the best to our knowledge only few studies have focused on STI patients [8]. STIs have become one of the most serious public health problems worldwide, with 500 million infections documented in 2016 [19]. Moreover, the prevalence of STIs in China is also on the rise, prominent among which is syphilis, with an incidence rate estimated at 32 per 100,000 population in 2016 [20]. And the most economic and effective way to prevent sexually transmitted infection is to advocate protected intercourse. Thus, in the current study, sexually active STI patients were included to investigate the correlation between SC and UI.

However, it is clear from some studies that the correlation between SC and UI does not apply to the entire target population, with differences in regular and casual sexual partners. As early as 2004, Kalichman found that while results showed increased rates of risk behaviors in people with higher SC overall, particular risks were found to be associated with casual and one-time sexual partners [8]. Another study targeting sexually active heterosexual Hong Kong Chinese males in 2015 found that among participants with causal sexual partners, a higher SCS score was significantly associated with UI, whereas such an association was not evident among those with regular sexual partners [10]. With complex and concealed nonmarital sexual behavior gradually becoming the main route of HIV transmission worldwide [21], many previous studies have taken note of this issue [5–7, 22, 23]. Another factor worth noting is demographic characteristics. As there are differences in gene, personality and gender in many behaviors [24–26], there are also significant differences in sexual impulsivity between men and women. A significant divergence exists, with men scoring evidently higher in SC than women [27–29]. This difference does not necessarily affect the correlation between SC and UI but is a reminder that, while targeting a population covering both genders, it is worth studying whether this difference is factored into each gender with regarding to UI.

The relationship between SC and UI is an unavoidable subject in STI patients, a population that is vulnerable to high UI prevalence and with the potential to further spread STIs. However, few studies focus on these patients and even fewer take the gender factor and type of sexual partner into consideration. To our knowledge, this is the first study that examine the following hypothesis in STI patients: (i) gender is an influencing factor of sexual compulsivity; (ii) SC can also act as a predictor of UI; and (iii) the correlation between SC and UI can vary in regard to intercourse with regular versus casual sexual partners.

## Methods

### Study site

Shanghai Skin Diseases Hospital affiliated to Tong Ji University School of Medicine, which was built in 1945, is the only public hospital that specializes in STIs in Shanghai. The STI Department of the hospital has established the only clinical research and basic scientific research platform for the study of neurosyphilis in China.

### Participants

Participants were receiving services from two selected STD (sexually transmitted disease) clinics (two branch divisions of Shanghai Skin Diseases Hospital on Baode Road and Qiujiang Road, both in the Jingan District) in Shanghai during June to December 2018. Those who met the following criteria were invited to participate: aged ≥18 years, clinically diagnosed with STIs, admitted having sexual congress in the past 6 months, and able to read the informed consent form. Patients with the following characteristics were excluded: unconsciousness, cognitive impairment or/and severe mental disability caused by neurosyphilis, serious audiovisual impairment or poor reading ability and/or unable to understand the study’s aims and contents, and reluctance to cooperate.

### Procedures

Our survey team signed cooperation agreements with the Shanghai Skin Disease Hospital before beginning the survey. All of the doctors who worked in the STD department (inpatient or outpatient) were recruited and were informed about the survey beforehand. The doctors then informed each participant about the survey before each interview. All interviewers were senior medical students and graduate students at the Shanghai Jiao Tong University School of Medicine who were trained before the interviews and had several in-person reviews throughout the study. The training also incorporated quality-control strategies, such as reexamining and investigating the questionnaires and resolving issues that may have arisen during the fieldwork. Anonymous face-to-face interviews were conducted with the participants in a separate room to protect their privacy and collect valid data via the questionnaire. Each participant received 80 RMB (approximately US$12) in cash for their participation.

### Ethics

The study was approved by the Shanghai Jiao Tong University School of Medicine Public Health and Nursing Ethics Committee. Written informed consent was provided by all participants before the interviews. All participants were free to ask any questions and to withdraw if they did not wish to continue.

### Measures

#### Sociodemographic variables

A questionnaire was used to collect basic information of the participants, including age, gender, sexual orientation, marital status, education level, monthly income, HIV status, diagnosis, case and sexual partners.

#### Sexual compulsivity variables

The 10-item SCS, derived from Kalichman’s questionnaire in 1994 (α = 0.88), was used to evaluate SC in this study [11]. The School of Public Health, University of Hong Kong, has translated this scale to produce a Chinese version, the validity of which has been evaluated by Liao Wei [10]. Responses are made on a four-point Likert scale, ranging from “very strongly disagree” (1 point) to “very strongly agree” (4 points). Total scores range from 10 to 40, with a higher score having a positive correlation with the degree of SC. In our study, the Cronbach’s α of SCS was 0.939.

#### Unprotected intercourse

The participants were asked whether they had UI or not during sexual activities with either regular or casual sexual partners during the past 6 months. Responses were recorded as a binary variable: participants either chose “yes” or “no” when answering “Did you use condoms every time during the past 6 months during sexual activities?” Participants who did not choose “yes” in both categories were deemed as having UI.

### Statistical analysis

Cronbach’s α was used to assess internal reliability. Mean and standard deviation were used for descriptive analysis. Between-group and within-group differences were compared, respectively, by *t* test and ANOVA. The associations between SCS score and UI were investigated using univariate and odds ratios (OR) with their respective 95% confidence intervals (95% CI). We entered the total score and the two subscale scores separately in three different models. In addition, multiple logistic regression models were fitted, adjusting for significant sociodemographic variables related to UI in the unadjusted model. Statistical significance was defined as *p* < 0.05, and IBM SPSS for Windows, Version 20.0 (IBM, Armonk, NY, USA) was used for all statistical analyses.

## Results

### Demographic characteristics

Table 1 showed the demographic characteristics of a total of 664 patients (302 males, 362 females), with the age of more than half (58.73%) falling within the range 26–40 years old. Regarding sexual orientation, 88.10% considered themselves heterosexual and 11.90% as sexual-orientation minority. Regarding education and marital status, 3.46% reported having a highest education of college degree or above while 56.93% reported having a highest academic qualification of primary school education or less; 63.4% reported being married. 24.15% of the sample earned more than 12,000 RMB (about US$1700) every month, and 11.90% reported their monthly income less than 3000 RMB (US$425). 449 of 664 participants (191 males and 258 females) reported had UI during the past 6 months. 67.17% of the participants reported that they only had regular sexual partners during the past 6 months. The only three variables-gender, sexual orientation, and sexual partners- were significantly associated with sexual compulsivity, with male’s score higher than females, sexual orientation minority’s score higher than heterosexual’s and those who had only casual sexual partners scored highest (see in Table 1).

**Table 1:** Demographic characteristics and relationships with sexual compulsivity among sexually active STI patients (*N* = 664)

Demographic characteristics	Number (*n*)	Percent (%)	*Score(x* ± SD)	*p*
Gender				
Male	302	45.48	20.45 ± 5.83	< 0.001
Female	362	54.52	17.35 ± 5.00	
Age				
≤ 25	86	12.95	18.77 ± 4.81	0.951
26–40	390	58.73	18.87 ± 5.45	
41–60	159	23.95	18.71 ± 6.21	
≥ 61	29	4.37	17.55 ± 6.43	
Sexual Orientation				
Heterosexual	585	88.10	18.52 ± 5.63	< 0.001
Sexual Orientation Minority	79	11.90	20.57 ± 5.11	
Education Level				
Primary Degree and Below	378	56.93	18.46 ± 5.55	0.227
Middle School Degree	145	21.84	19.55 ± 5.50	
High School Degree	118	17.77	19.93 ± 6.01	
College Degree and above	23	3.46	17.35 ± 4.67	
Marital Status				
Single	200	30.12	18.81 ± 4.94	0.989
Married	421	63.40	18.77 ± 5.98	
Divorced	31	4.67	18.55 ± 4.28	
Widowed	12	1.81	18.08 ± 6.01	
Monthly Income				
≤ 3000 RMB	79	11.90	18.32 ± 6.09	0.376
3001–6000 RMB	218	32.83	19.00 ± 5.37	
6001–12,000 RMB	200	30.12	18.78 ± 5.60	
≥ 12,001 RMB	167	25.15	18.63 ± 5.71	
HIV Status				0.702
Positive	65	9.79	18.98 ± 5.60	
Negative	351	52.86	18.91 ± 5.65	
Unknown	248	37.35	18.50 ± 5.56	
Diagnosis				0.802
Genital warts	277	41.72	19.07 ± 5.61	
Syphilis	239	35.99	18.80 ± 5.86	
Gonorrhea	26	3.92	18.15 ± 5.37	
Genital herpes	23	3.46	18.13 ± 4.18	
Others	99	14.91	18.12 ± 5.33	
Case				
Outpatient	477	71.84	18.69 ± 5.52	0.541
Inpatient	187	28.16	18.95 ± 5.83	
Had UI during the past 6 months				
Yes	449	67.62	18.74 ± 5.55	0.782
No	215	32.38	18.82 ± 5.73	
Sexual Partners during the past 6 months				
Regular Sexual Partners	446	67.17	18.05 ± 5.34	< 0.001
Casual Sexual Partners	71	10.69	21.14 ± 5.90	
Both Regular and Casual Sexual Partners	147	22.14	19.77 ± 5.81	

### Binary regression coefficients of sexual compulsivity and unprotected sex

To corroborate the statistical data, we used continuous variables instead of cutoffs to analyze the difference between male and female gender regarding the association between SC and UI. Table 2 showed that a statistical difference was found in UI with a casual sexual partner, whereas a correlation between SC and UI was not evident with regular sexual partners. Moreover, no correlation was found among female participants. Thus, there is a clear difference between male and female participants, and between different types of sexual partner.

**Table 2:** Binary regression coefficients of sexual compulsivity and unprotected intercourse

Dependent variables	Male		Female		Total	
	OR (95%CI)	AOR (95%CI)	OR (95%CI)	AOR (95%CI)	OR (95%CI)	AOR (95%CI)
Unprotected intercourse with either regular sex partner or casual partner	1.009 (0.969–1.050)	1.009 (0.969–1.050)^a^	0.973 (0.929–1.019)		1.003 (0.974–1.032)	1.000 (0.971–1.031)^g^
Unprotected intercourse with regular sex partner	0.974 (0.936–1.013)	0.794 (0.936–1.014)^b^	1.007 (0.964–1.052)	1.010 (0.954–1.070)^d^	0.976 (0.950–1.004)	0.980 (0.952–1.008)^e^
Unprotected intercourse with casual sex partner	1.077 (1.026–1.130)^*^	1.079 (1.028–1.133)^*,c^	1.030 (0.976–1.087)		1.062 (1.026–1.099)^*^	1.058 (1.021–1.096)^*,f^

^a^Adjusted odds ratio (AOR) adjusting for degree, sexual orientation, age

^b^AOR adjusting for degree, marital status, sexual orientation

^c^AOR adjusting for degree, age

^d^AOR adjusting for degree, marital status, sexual orientation, age

^e^AOR adjusting for degree, sexual orientation

^f^AOR adjusting for degree, marital status, sexual orientation, age

^g^AOR adjusting for degree, sexual orientation, age

*p < 0.01

## Discussion

The results of our study demonstrated that SC did not correlate with UI among all 664 surveyed STI patients in Shanghai. However, SC was evidently a potential predictor for UI with casual sexual partners, especially among the male participants.

To our knowledge, this is the first research to focus on sexually active STI patients on mainland China, a population vulnerable to HIV and other STIs. The results demonstrated a difference between male/female gender and regular/casual sexual partners. Not surprisingly, greater sexual preoccupations and poorer control of sexual impulse were associated with a greater possibility of UI with casual sexual partners. The same result has also been reported for other sexually active populations, including homosexual males, lesbians, bisexual men and women, and HIV-positive populations [10, 16, 30]. In accordance with all previous studies [27–29], we found that the score of SC was significantly associated to gender, with men scoring higher than women. While SC predicts the possibility of UI with a casual sexual partner among males, the association is not as obvious in females. Moreover, a significant difference also exists between regular and casual sexual partners. SC seemed to correlate only with increased UI in casual rather than regular sexual partners.

These two differences are the most intriguing takeaways from the results of this study. That female SC does not seem to directly affect the incidence of UI could be due to the fact that women tend to be in a disadvantaged position when it comes to applying protection. Female condoms are not as common and easily accessible as male condoms [31, 32], and persuading a casual sexual partner who is reluctant to use protection can be difficult. Men will have more control in this situation because their willingness to use condoms can be more directly transformed into action.

The difference between regular and casual sexual partners was also found in several previous studies among other sexually active populations [8, 10, 33], one of which was conducted in China [10]. It could also be possible that higher SC results in more intercourse with casual partners, which is a less controllable scenario in comparison with regular partners, thus resulting in greater possibility of UI [34]. Chinese culture has a tendence to discourage people from expressing sexual drive and suppress their sexual compulsivity, especially in front of people who are supposed to have a secularly appropriate relationship [35]. Meanwhile, the Internet use becomes an available tool which ‘opens the world’ to seek casual sexual partners [36]. Thus, it can be easily understood why our participants who had higher SC no longer seek sexual fulfillment with regular sexual partners but instead freely express their SC while engaging with casual partners.

According to the theory of planned behavior, perceived behavioral control is one of the most important predictors of an individual’s intention and behavior [37, 38]. Evidence has shown that less perceived behavioral control over condom use is correlated with practicing UI. People who have poor impulse control also tend to have a higher anxiety and depression level [39–41], which could result in more risky behavior. The high prevalence of UI and the fact that our participants already had STIs together lead to a higher risk of acquiring HIV and other STIs as well as acting as a bridge to spread the diseases, although compared with university students, unmarried youth, migrant workers, and sex workers, STI patients are more likely to have already received more health education on this matter, possibly due to their familiarity with the diseases, the fear of progression, and contact with clinicians [42]. The prevalence of UI is still high, which may be associated with the patients’ innate high SC in their personality.

The overall prevalence of sexual preoccupation and poor impulse control in Shanghai STD clinic cohorts supports a susceptibility for STIs among people of similar demographic characteristics with persistent and uncontrolled sexual thoughts and impulses. Traditional models of public health education and behavioral interventions will likely prove insufficient and ineffective for this population [43]. If validated through further research, these STI results indicate an urgent need for preventive interventions targeted toward people who lack control of sexual thoughts, behaviors, and impulses.

General health education may work poorly in this population because the overall education level was shown to be low, with only 3.46% reporting a highest education of college degree or above while 56.93% reported having a highest academic qualification of primary school level. Therefore, we urgently need to design new ways to intervene [44]. The most promising intervention models for this population may be those that integrate elements of mental health, treatment of substance abuse, and reduction of sexual risk. For example, behavioral self-management approaches used in cognitive behavioral therapy for sexual preoccupations and poor impulse control can be adapted for inclusion in STI risk-reduction counseling [7]. Public health clinics should also be prepared to refer their clients who express distress about feeling uncontrolled sexual desire and behavior for help that goes beyond services that an STD clinic can provide [45]. STD clinics in Shanghai can work with psychiatric hospitals and establish a system that can effectively help the patients to manage their sexual impulse.

Furthermore, the difference we found between males and females can provide a new approach to the prevention of UI that would include two different intervention procedures appropriate for males and females, respectively [46]. If women’s disadvantaged position in applying protection prevents them from having protected intercourse according to their own will, we should establish a method that can somehow shift that disadvantage [47–49]. For example, STD clinics can recommend that their female patients prepare female condoms themselves so that they can be less passive when the situation arises. Also, health education can place more emphasis on the importance of having control in these situations, and how to communicate with the other party to achieve protection or refuse if the sexual partner cannot be persuaded, thus combining the management of SC with custom health education for each gender. Meanwhile, the reported difference between regular and casual sexual partners [50–53] suggests that we should focus on the subpopulation with more casual partners when further studying this subject, not only because they show a stronger correlation between SC and UI but also because having more casual sexual partners equates to a higher possibility of spreading STIs and HIV [1, 2, 8]. It is hoped that a more efficient and promotable method can be found, resulting in the reduction of risky sexual behavior and, ultimately, reduction of the prevalence of STIs and HIV in Shanghai.

## Conclusion

This study extends the literature in several important ways. We demonstrated that SC was evidently a potential predictor for UI with casual sexual partners among sexually active STI patients, especially the male participants. More attention should be paid to implementing unique and targeted behavioral interventions among highly sexually compulsive STI patients. In particular, more attention should be paid to patients with more frequent sexual behavior with casual partners. In addition to general sexual education, counseling interventions should also be provided by other health institutions, and specific intervention methods targeting each gender should be considered among the sexually active STI patients, which, it is anticipated, will help reduce risky sexual behavior among STI patients, resulting in overall reduction of the prevalence of STIs and HIV in Shanghai.

Several limitations should be considered in interpreting the present results. First, cross-sectional surveys have difficulty determining causality; therefore, a prospective study would be beneficial. Second, although this research is a double-center cross-sectional study in a hospital specializing in STDs, the sample size was not especially large, so more multicenter research is needed. Third, there was selection bias: for example, those who experienced strong SC but felt bad about partaking in casual sexual activities may also have been more reluctant to participate in the study. Fourth, a self-reported binary scale was used to assess SC and UI, which potentially underestimated their prevalence. Also, the present study only focused on sexually active STI patients rather than the general STI population. Further follow-up research should target other high-risk groups or study whether the SCS can be used to identify healthy people who are prone to UI and STDs, and to intervene in advance. Considering there are large number of migrants in Shanghai, whether married STI patients are living with their spouses should be investigated in future research. Moreover, the relationship between SC and crime also needs to be examined.

## Data Availability

The datasets used and/or analysed during the current study are available from the corresponding author on reasonable request.

## Abbreviations

STI: Sexually transmitted infection
UI: Unprotected intercourse
SC: Sexual compulsivity
HIV: Human immunodeficiency virus
SCS: Sexual compulsivity scale
STD: Sexually transmitted disease
OR: Odd ratio
AOR: Adjusted odd ratio
CI: Confidence interval
MSM: Men who have sex with men
